# The β-Cell Genomic Landscape in T1D: Implications for Disease Pathogenesis

**DOI:** 10.1007/s11892-020-01370-4

**Published:** 2021-01-02

**Authors:** Mireia Ramos-Rodríguez, Beatriz Pérez-González, Lorenzo Pasquali

**Affiliations:** Endocrine Regulatory Genomics, Department of Experimental & Health Sciences, University Pompeu Fabra, 08003 Barcelona, Spain

**Keywords:** Type 1 diabetes, Beta cells, Pancreatic islets, Regulatory genomics, Human genetics, Epigenomics

## Abstract

**Purpose of Review:**

Type 1 diabetes (T1D) develops as a consequence of a combination of genetic predisposition and environmental factors. Combined, these events trigger an autoimmune disease that results in progressive loss of pancreatic β cells, leading to insulin deficiency. This article reviews the current knowledge on the genetics of T1D with a specific focus on genetic variation in pancreatic islet regulatory networks and its implication to T1D risk and disease development.

**Recent Findings:**

Accumulating evidence suggest an active role of β cells in T1D pathogenesis. Based on such observation several studies aimed in mapping T1D risk variants acting at the β cell level. Such studies unravel T1D risk loci shared with type 2 diabetes (T2D) and T1D risk variants potentially interfering with β-cell responses to external stimuli.

**Summary:**

The characterization of regulatory genomics maps of disease-relevant states and cell types can be used to elucidate the mechanistic role of β cells in the pathogenesis of T1D.

## Introduction

Type 1 diabetes (T1D) is the most common form of diabetes in childhood, accounting for approximately 75% of diabetes new diagnoses in patients ≤ 19 years of age. In North America and Europe, the incidence of T1D varies between 4 and 41 per 100,000, with approximately > 17,000 new cases occurring annually in the USA [1–3].

T1D is a chronic autoimmune disease that develops from the interaction of genetic and environmental factors [4]. Combined, these factors trigger an aggressive autoimmune assault against pancreatic β-cells, provoking local inflammation of pancreatic islets (insulitis) and progressive loss of β-cells [5, 6]. Genetic and non-genetic factors likely operate at all stages of this process [7]. Currently, our knowledge about the molecular mechanisms linking genetic variation and environmental triggers with T1D remains limited.

The disease develops upon inheritance of an adaptive immune system genetically prone to respond to β-cell antigens [8]. In this context, autoreactive T cells that have escaped central tolerance drive the destruction of β-cells through cytokine-mediated mechanisms and direct human leukocyte antigen (HLA) class I cytotoxic killing [9]. Growing evidence points to an intrinsic, genetically determined, β-cell vulnerability to cell death, as a central mechanism driving T1D pathogenicity. Understanding the genetic basis of β-cell vulnerability in T1D holds the promise to solve critical disease mechanisms that could, in turn, be the target of new therapeutic approaches.

These review overviews research deciphering disease mechanisms using genetic approaches with a focus on the implication for the β-cells in T1D pathogenesis.

## Deciphering the Genetic Component of T1D

The exact mechanisms that trigger the β-cell-targeted autoimmune attack are not fully understood. In line with a multifactorial aetiology, T1D presents a non-Mendelian inheritance pattern, showing a strong heritable component supported by up to 70% twin concordance [10] and sibling risk around 8% [11, 12].

In monogenic diseases, DNA sequencing has greatly improved clinical practice and genetic counselling through the identification of causal genes. This advance has in turn helped to clarify the disease pathogenesis, thus, opening the path to the development of new treatments or the use of etiologically directed drugs [13]. In complex or multifactorial diseases, the pathway to personalized medicine is more challenging and requires deciphering of a complex genetic architecture underlying broad and heterogeneous clinical spectra [14]. The detection of genetic association signals, often driven by common low-impact risk variants, offers the opportunity to gain mechanistic insight into the disease pathogenesis and shed light on the key genes implicated in the development of T1D.

Additionally, trait-associated genetic variants may be useful to identify individuals who are at higher risk of developing a specific disease. This concept underlies the development of polygenic risk scores (PRS), used to describe the overall genetic risk of a given individual, based on the combination of all variants, including genetic signal with modest effect size, associated to a disease [15]. Thus, each variant is weighted according to its contribution and effect direction [16]. In T1D, PRS offers the opportunity of using disease prediction for a more meticulous surveillance of at-risk individuals or for contemplating their inclusion into trials of early immunological intervention [7]. As an example, Sharp et al. [17] successfully developed a PRS based on 67 different SNPs, including both HLA DR-DQ haplotypes and non-HLA variants. This study allowed discrimination of T1D candidates with outstanding accuracy, performing 50% better than other PRS in T1D prediction when applied to general population. However, most genetic studies are performed in populations with European ancestry, leading to an increased risk of misdiagnosing or misclassifying in underrepresented populations, as the transferability of the same PRS between populations is low [12, 18].

The strong commitment of families with affected members to genetic research efforts has allowed to gain insights into the genetic architecture of T1D and, to some extent, to develop disease prediction based on patient’s genetic background [7].

### T1D Genetic Risk Factors

During the last decades, large efforts have been dedicated to genotyping and analysis of thousands of human genomes with the aim of finding DNA variants associated with complex diseases as well as common traits. These studies, known as GWAS (genome-wide association studies), have revealed more than 60 regions that influence T1D risk, explaining almost 80% of disease heritability [19, 20].

As for other complex diseases, the overall influence of the genetic background in T1D results from the contribution of genomic variants that (with the remarkable exception of the human leukocyte antigen (HLA) region) individually exert a modest effect and have a small impact on the clinical outcome. While HLA-associated variants were the first to be reported [21] and show the highest impact on T1D risk [22], non-HLA loci include common, low-impact associated variants, possibly affecting the disease progression and the speed at which functional β-cell mass is lost [23, 24].

#### HLA Loci

The highly polymorphic HLA region on chromosome 6p21 encodes for glycoproteins belonging to the major histocompatibility complex (MHC). These molecules interact with peptide antigens, allowing the immune cells to recognize non-self antigens and trigger an immune response. The HLA region is strongly associated with T1D, accounting for approximately 50% of the overall heritability [25].

HLA class II genes (HLA-DP, HLA-DQ and HLA-DR) code for proteins expressed by antigen-presenting cells (APCs). Approximately, 90% of T1D cases present HLA class II risk haplotypes with variants conferring the strongest susceptibility being located at highly polymorphic sites, encoding for the peptide-binding pockets of the DQ and DR molecules [26, 27]. For example, in Caucasians, common haplotypes conferring susceptibility to T1D are those that encode for the DR4/DQ8 and DR3/DQ2 molecules, thus associating HLA-DQ2/DQ8 with higher risk of T1D (with an odds ratio of almost 11). On the other hand, another haplotype in this region, coding for the DQ6 molecule, is associated with a protective role [12].

In addition to HLA class II risk variants, genetic association to T1D was also found at the HLA class I region, particularly in HLA-A and HLA-B [26, 27]. Classical HLA class I genes (HLA-A, HLA-B and HLA-C) encode for proteins that have a critical role in cytotoxic CD8^+^ T cell-mediated activity, the main mechanism of T1D-associated autoimmunity. This locus also contains other non-classical HLA class I genes, such as HLA-E, which encodes for proteins involved in antigen presentation to natural killer (NK) cells [28, 29]. It is likely that risk alleles at these genomic loci influence β-cell destruction and T1D progression. Indeed, HLA-A*24 allele is associated with low residual β-cell function, possibly due to an enhanced immune-mediated destruction of insulin-producing cells in individuals carrying the risk haplotype. Other examples of variants in class I HLA genes that modulate T1D genetic risk in both directions have been reviewed in more detail elsewhere [22, 29].

#### Non-HLA Loci

The first non-HLA genetic signals associated with T1D were obtained from small candidate gene association studies and family linkage analyses. Such studies often had small sample sizes and were thus underpowered, but allowed to identify several key candidate genes outside the HLA locus.

The 5′ upstream region of the insulin locus (*INS*) is the second genomic region with strongest association with T1D risk (about 10% contribution to T1D susceptibility) [23] and the first non-HLA locus to be associated with T1D [30]. In particular, most genetic association comes from the variable number of tandem repeats in this locus (INS-VNTR). The VNTR alleles are classified according to their length, and shorter alleles (class I) have been associated with an increased risk of T1D by influencing the expression of (pro)insulin in the thymus. This is an example of an allele-specific mechanism that helps to capture the complexity of T1D-associated genetic risk [31]. In contrast, long VNTR alleles (class III) seem to confer protection against T1D, as they were shown to promote higher levels of insulin transcription in the thymus during the induction of central immune tolerance [23, 32, 33].

The *CTLA4* locus has also been associated with T1D, although this relationship varies across different ethnic populations. CTLA4 is a known extracellular receptor that is a negative regulator of cytotoxic CD8^+^ T cell immune responses [34–36]. Additional loci initially associated to T1D prior to application of GWAS include protein tyrosine phosphatase, non-receptor type 22 (*PTPN22*) gene [37, 38], interleukin 2 receptor alpha (*IL2RA*) [39, 40], ubiquitin-associated and SH3 domain-containing protein A (*UBASH3A*) [41] and interferon-induced helicase c domain-containing protein 1 (*IFIH1*) [33, 42].

The application of GWAS together with their meta-analyses allowed the identification of novel non-HLA regions associated with T1D risk [43–47]. Such studies represented an important effort of the scientific community and are key to elucidate the genetic background of T1D, as they provide precious information to unravel the molecular mechanisms that underlie the disease pathogenesis. Major opportunities may result from the challenging interpretation of the data arising from GWAS, including (1) translation of the genetic signals associated with T1D into molecular mechanisms central to the disease pathogenesis, (2) detection of the causative risk variants and the dissection of their individual contribution to the disease and (3) translation of the aggregate genomic variation linked to the disease trait into tools that may assist individualized patient clinical management [18].

Latest novel non-HLA loci findings include the T1D-centered association study by Onengut-Gumuscu et al. [48], in which they used ImmunoChip, a SNP microarray-centred on immune-disease-region, to unmask more than 40 non-HLA associated loci, 4 of which were novel associations. More recently, the same platform was applied to the currently largest and most ancestrally diverse genetic T1D cohort including 61,427 participants and yielding to 36 novel additional regions associated to genome-wide significance [49].

For some non-HLA T1D risk loci, a candidate gene has been identified, including the autoimmune regulator (AIRE) transcription factor, which is highly expressed in the thymus where it is implicated in the development of tolerance to self-antigens [50, 51]. Other candidate genes are protein tyrosine phosphatase non-receptor type 2 (*PTPN2*), interleukin genes (such as *IL4*, *IL13*, *IL4R*, *IL10*), insulin receptor substrate 1 (*IRS-1*), inducible T cell costimulator (*ICOS*) and small ubiquitin-like modifier 4 (*SUMO4*), which modulate susceptibility to T1D or disease progression [12, 23].

Nonetheless, one of the main current challenges includes that of identifying the target gene or genes at each GWAS-associated region and unravelling how their alleles affect downstream phenotypes. Such effort could in turn allow gaining deeper understanding of the mechanisms underlying T1D pathology and assess new druggable targets.

### Genetic Variants and the Non-coding Genome

Most but not all disease-associated SNPs are found outside protein-coding sequences, mostly localized in at intergenic regions [12, 18, 19, 29], suggesting that T1D-associated genetic variation may impact regulatory functions rather than affecting the gene-coding potential. Genomic regulatory functions are highly dynamic and cell type-specific. Thus, identification of causal regulatory variants requires knowledge of tissue- and state-specific regulatory landscape of the cell types implicated in T1D pathogenesis [19].

During the last decades, the effort of single laboratories and of large consortia such as ENCODE and the Epigenome Roadmap, resulted in detailed annotation of the non-coding regions of the human genome for a large number of human tissues, including several relevant to T1D. Integration of such regulatory maps with GWAS data from different traits is pivotal to translate genetic association signals into molecular mechanisms [52]. Variants associated to complex diseases, including T1D, were found to overlap regulatory elements more than expected, including tissue-specific enhancers [19, 53, 54]. Fine mapping was subsequently used to prioritize disease-associated variants by integrating the association signal with genomic information such as gene expression, transcription factor-binding sites, DNA methylation, histone modifications or open chromatin regions. For a number of genetic association signals and different traits, these studies allowed the identification of functional risk variants affecting the tissue-specific regulatory code [18]. To date, there are dozens of examples of GWAS-associated variants affecting regulatory functions in different cell types and disease traits, including some in T1D [48, 55, 56]. As an example, one recent study [56] focused on a GWAS signal located at human chromosome 11q13.5 and shared by several autoimmune diseases, including Crohn’s disease, ulcerative colitis, T1D and asthma. Integration of the genetic signal with cell-specific regulatory maps revealed that several associated variants in the locus interfere with the activation of a CD4^+^ regulatory T cell distal enhancer that induces the expression of *LRRC32* and is required for T cell-mediated suppression of colitis.

Information on the genes affected by T1D regulatory variants is crucial to the development of therapeutic interventions targeting the disease pathogenic mechanisms. While regulatory maps allow prioritizing disease-associated GWAS variants falling in the non-coding genome, identification of the target gene(s) requires resolving regulatory relationships at the associated regions.

Most GWAS loci associated to a trait are conventionally named after the gene(s) with putative biological significance in linear proximity to the leading SNP (a variant in the locus showing the strongest association) of the region. Nevertheless, studies attempting to link distal regulatory elements to their target genes show that linear proximity provides a poor prediction. For example in one study, combination of chromatin capture and genome-editing techniques revealed that using distance in linear DNA as the only metric to link a gene promoter to a disease-relevant distal enhancer was not predictive of the correct target gene(s) for more than 70% of the T2D-associated loci [57].

Therefore, the study of the 3D chromatin architecture is one approach that offers precious information on the gene targets at GWAS susceptibility loci, as regulatory elements are thought to physically interact with their target gene(s) to regulate gene expression. However, it remains largely unknown whether risk variants interfere with chromatin looping, leading to changes in gene regulation. A recent study generated high-resolution 3D chromatin maps in immature thymic T cells of mice with T1D predisposition [58]. The authors found that chromatin folding was altered at T1D risk-conferring loci, resulting in 3D chromatin interaction changes when compared to control mice. Moreover, they found that alterations in 3D genome architecture lead to gene expression changes in pancreas-infiltrating immune cells from T1D patients. Such observations indicate that T1D pathogenesis may involve changes in the 3D chromatin structure, likely altering regulatory interactions that lead to gene expression changes.

### Cell Types Implicated in T1D Pathogenesis

The pathogenesis of complex diseases such as T1D typically implicates multiple cell types [18]. Classically, T1D GWAS variants have been mostly considered to impact the immune system. This hypothesis is in line with the autoimmune nature of the disease and is supported by multiple studies showing that T1D risk variants are enriched in enhancers active in CD4^+^ and CD8^+^ T cells [48, 49, 59]. Farh et al. [59], by integrating autoimmune disease GWAS data with regulatory genomic annotations, found that ~ 90% of risk variants accumulate in non-coding regions. While 60% of these SNPs map to immune-cell enhancers, the authors reported only a reduced number of T1D variants overlapping islet enhancers. Similarly, Onengut-Gumuscu et al. [48] found a strong enrichment for T1D risk variants to overlap regulatory elements active in immune cell types, but not for pancreatic islet enhancers.

Work from other autoimmune diseases highlights that the cell types in which the risk allele exerts a pathogenic effect may include other non-immune cell types. By applying an alternative approach on a locus-by-locus basis, rather than computing conventional enrichment strategies, Factor et al. [60] uncovered several risk loci acting in oligodendrocytes and inducing an alteration of myelin production in the context of the pathogenesis of multiple sclerosis. These results do not contradict the current knowledge regarding the role of the immune system in disease aetiology. Instead, this work provides a better understanding of the disease pathogenesis and may help the development of more effective therapeutic approaches.

While it is now well established that alterations to the immune system are key in T1D pathogenesis, the concept that β-cells actively contribute to the development of the disease has gained traction during the last few years.

In support of this hypothesis, β-cell endoplasmic reticulum (ER) stress was shown to be present in T1D patients [61] and to temporally precede the development of hyperglycaemia in the T1D NOD mouse model [62]. Activation of these pathways has been observed in conjunction with β-cell HLA class I overexpression, which in turn serves as signal for immune-mediated β-cell destruction [63, 64]. Furthermore, cytokine-induced stress is coupled with the production of immunogenic peptides able to trigger or amplify the immune response [5, 65, 66] .

Such pathways may as well be activated upon cellular senescence. In T1D patients and NOD mice, a subset on β-cells was shown to acquire senescence-associated phenotype. Importantly, clearance of those senescent β-cells preserves β-cell mass and reduces diabetes incidence in mouse models [67].

Indeed, recent studies, described in the following sections, suggest that several T1D risk alleles may exert a pathogenic effect by interfering with β-cell regulatory networks and their response to an inflammatory environment [7, 20, 55].

## T1D Risk and β-Cell Non-coding Functions

During the past few years, the accumulating evidence for an active role of β-cells to T1D pathogenesis resulted in the engagement of different laboratories in attempting to map disease-associated variants to regulatory regions active in β-cells, as a means to characterize the mechanistic role of β-cells in T1D development.

### Regulatory Maps of Pancreatic Islets in Resting Conditions

Human pancreatic islet regulatory landscape has been linked consistently to T2D risk, since islet regulatory elements were shown to be enriched in T2D disease variants [57, 68–70]. In T1D, some studies reported limited overlap of disease-associated variants with islet regulatory elements [59]. However, most of the T1D genetic association signal coincides with regulatory elements active in immune cell populations [48, 59].

Being that the β-cell failure is a central event to the pathogenesis of both T1D and T2D, several research groups interrogated pancreatic islets regulatory networks in search of shared genetic contributions to the risk of developing the diseases. Such effort is in line with the “β-cell fragility” model, which proposes that a genetically determined increased risk of β-cell death or insulin secretion dysfunction may contribute to the risk of developing either T2D or T1D, especially in the presence of immunological and/or metabolic stress factors [71–74].

Aylward et al. [75] observed a shared genetic risk mostly involving variants affecting islet function and insulin secretion. Interestingly, the authors uncovered a candidate-shared variant located in the proximity of the gene *GLIS3*, which mapped to a pancreatic islet accessible chromatin site and showed allele-specific enhancer activity in mouse β-cells. Similar findings at the *GLIS3* locus, although associating different variants, were observed in a study applying stringent statistical methods in a large cohort of patients, also aiming to uncover co-localization of T1D and T2D genetic signals. In this study, the authors extend their findings to four additional regions in proximity of the genes *PGM1*, *TMEM129*, *INS* and *BCAR1/CTRB1* [76]. Unlike for *GLIS3*, which shows concordant direction of effect, for these four additional signals, the effect of genetic variants in T1D and T2D was in opposite directions. Such important observations suggest that genetically identified drug targets would have exclusive efficacy in the treatment of one of the two diseases, making it key to find proper diagnostic tools to avoid misdiagnosis in older individuals.

Further insights into the role of *GLIS3* and other T1D risk loci in pancreatic islets were provided by Inshaw et al. [77]. In this study, six HLA and six non-HLA risk alleles were associated with stronger effect sizes in T1D patients under 7 years of age, compared to ≥ 13-year-old patients. The authors highlight the candidate gene *GLIS3* to likely act through β-cells and *CTSH* and *IKZF3*, which may act through pancreatic islets or other tissues. A T1D-associated variant at the *CTSH* locus (15q25.1) was shown to co-localize with a *CTSH* eQTL in which the risk allele is associated with the upregulation of the transcript.. An LD block of 103 variants was prioritized at the *IKZF3* locus (17q12-q21.1) although further analyses, such as intersection with regulatory maps, are needed to identify functional variants. IKZF3 is a transcriptional repressor already implicated in asthma and other autoimmune diseases. Interestingly, the risk allele seems to exert an opposite direction-of-effect, as it increases susceptibility to autoimmunity but protects against asthma.

In conclusion, several studies uncovered examples of T1D variants acting through regulatory elements active in unchallenged pancreatic islets. Nevertheless, the number of potentially functional variants detected is relatively reduced when compared to studies showing that > 80% of T1D genes are expressed in pancreatic islets [78–80]. Such observation could in part be reconciled by expanding the repertoire of islet regulatory elements by charting state-specific regulatory maps in islet exposed to T1D-relevant stimuli.

### Regulatory Maps of Pancreatic Islets Exposed to External Stimuli

The hypothesis that chromatin maps were static and cells responded to environmental changes through a pre-established set regulatory elements was challenged by Ostuni et al. [81], who instead showed that new regulatory elements, which they called latent enhancers, could appear in adult macrophages in response to external stimuli. In β-cells, some studies have revealed changes in their regulatory landscape after exposure of human islets to external stimuli [55, 82].

During the first phase of T1D, β-cells are exposed to a proinflammatory environment. It is thus logical to reason that such environment may affect the β-cell cis-regulatory landscape. Indeed, Ramos-Rodríguez et al. [55] observed significant chromatin remodelling in human islets and β-cells treated with IFN-γ + IL-1β. Exposure to these proinflammatory cytokines revealed a set of cytokine-induced regulatory elements which they named induced regulatory elements (IREs). Moreover, the activation of novel regulatory elements was coupled with the establishment of new enhancer-promoter contacts linking IREs to their putative target genes. In turn, IREs were associated with the upregulation of nearby genes and their corresponding proteins, which are involved in immune response and pathways implicated in T1D pathogenesis. Importantly IREs were shown to be enriched for T1D risk SNPs. At 9 associated loci, T1D risk variants were found to directly overlap an IRE, suggesting that they might interfere with the pancreatic islet response to a proinflammatory environment. In line with this hypothesis, the risk alleles of rs78037977 (*FASLG-TNFSF18* region) and rs193778 (*CIITA-DEXI-CLEC16-SOCS1* region) T1D risk variants were shown to affect in vitro IRE enhancer activity in human β-cells exposed to proinflammatory cytokines.

Using a similar approach, Colli et al. [83] studied early insulitis by exposing β-cells to IFN-α. Similarly, IFN-α-induced chromatin remodelling was associated with upregulation of nearby genes and their corresponding protein. The study of the IFN-α-induced transcription factor binding sites induced by IFN-α exposure uncovered IRF and STAT as key drivers of the interferon signature in β-cells. Mining regulatory networks of the upregulated genes revealed two potential therapeutic interventions for reversing IFN-α deleterious effects on β-cells.

Both abovementioned studies take advantage of human pancreatic islets and a human β-cell line (EndoC-βH1 [84]), to characterize β-cell responses to T1D-relevant environmental changes. However, models have limitations: (1) exposure to proinflammatory cytokines represent an over-simplified in vitro model of an inflammatory environment which is far from mirroring the in vivo occurrence of insulitis, (2) gene regulatory responses in the β-cell line might differ from that of the primary tissue and (3) studying responses in human pancreatic islets provides challenges when attempting to dissect the β-cell contribution to the observed regulatory changes.

Nevertheless, such observations suggest that T1D-associated variants may act at a β-cell level in response to perturbations relevant to the disease pathogenesis (ex. inflammation), uncovering a novel potential mechanism linking genetic risk to T1D pathogenesis.

## Conclusions

Understanding the genetic basis of T1D is key to provide new clinical tools for patient management, as well as to shed light on the disease pathogenesis in an effort of identifying new etiologically driven drugs.

To understand the implication of T1D-associated variants to the disease pathogenesis, it is essential to unravel their target cell type(s) and their state. While many T1D-associated SNPs were shown to impact the immune system, several studies now reveal that a subset of T1D risk variants may act at the β-cell level, especially upon perturbations such as exposure to a proinflammatory environment. Such initial observations may be expanded by studying β-cell responses to a breath of disease-relevant conditions to fully characterize the pathology of T1D. Moreover, regulatory maps obtained from β-cells at different developmental stages or during senescence could be used to prioritize risk variants and reveal new insight into the pathogenesis of T1D and its genetic predisposition. β-cell enhancers containing T1D risk variants may also be active in other disease-relevant cell populations, such as T cells, thus making more difficult the dissection of the disease mechanisms.

Given the difficulty to access primary tissues from T1D patients in different T1D stages, in vitro models provide a key resource to decipher the role of β-cells in their own demise. However, such studies have to be considered with caution, as they use models that might not fully resemble the actual disease course. Current efforts to collect and study human islets from T1D patients, such as nPOD (Network for Pancreatic Organ Donors with Diabetes) consortia [85], may help to confirm and further investigate β-cell role in T1D. This, together with the advent of single-cell techniques, may allow improving the characterization of the islet cell populations through which T1D risk variants might be acting.

Shedding light on the genetic basis of β-cell fragility may open the path to solve critical disease mechanisms that could, in turn, be target of new therapeutic approaches.
